# Socioeconomic inequalities in lipid and glucose metabolism in early childhood in a population-based cohort: the ABCD-Study

**DOI:** 10.1186/1471-2458-12-591

**Published:** 2012-08-01

**Authors:** Gerrit van den Berg, Manon van Eijsden, Tanja G M Vrijkotte, Reinoud J B J Gemke

**Affiliations:** 1 Department of Pediatrics, VU University Medical Center, Amsterdam, The Netherlands; 2 Department of Epidemiology, Documentation and Health Promotion, Public Health Service of Amsterdam (GGD), Amsterdam, The Netherlands; 3 Department of Health Sciences, VU University, Amsterdam, The Netherlands; 4 Department of Public Health, Academic Medical Center, University of Amsterdam, Amsterdam, The Netherlands

## Abstract

**Background:**

Socioeconomic inequalities in cardiovascular disease are pervasive, yet much remains to be understood about how they originate. The objective of this study was to explore the relations of socioeconomic status to lipid and glucose metabolism as indicators of cardiovascular health in 5–6 year olds. Additionally to explore the explanatory role of maternal factors, birth outcome, and child factors.

**Methods:**

In 1308 5–6 year old ethnic Dutch children from the ABCD cohort study, lipids (cholesterol, LDL, HDL, triglycerides), glucose and C-peptide were measured after an overnight-fast.

**Results:**

There were no differences in cholesterol, HDL, LDL, and triglycerides between socioeconomic groups, as indicated by maternal education and income adequacy. However, children of low educated mothers had on average a higher glucose (*β* = 0.15; 95% confidence interval (CI) 0.03 – 0.27), logC-peptide (*β* = 0.07; 95% CI 0.04 – 0.09), and calculated insulin resistance (HOMA-IR) (*β* = 0.15; 95% CI 0.08 – 0.22) compared to children of high educated mothers. Only childhood BMI partly explained these differences (models controlled for age, height, and sex).

**Conclusions:**

The socioeconomic gradient in cardiovascular risk factors seems to emerge in early childhood. In absence of underlying mechanisms these empirical findings are relevant for public health care and further explanatory research.

## Background

Although socioeconomic inequalities in cardiovascular disease (CVD) are widely recognized, [1,2] the process by which the socioeconomic environment interacts with CVD remains unclear. Since there is increasing evidence that CVD originates in childhood and childhood socioeconomic status may have a persisting interaction with CVD in adult life, [3] it is mandatory to explore the socioeconomic gradient on the occurrence of cardiovascular disease associated risk factors in children.

Early cardiovascular risk factors can be identified in childhood. In a Danish study, there was a positive association between CVD in adulthood and an increasing body mass index (BMI) in childhood [4]. In addition, children with a higher BMI were more likely to have insulin resistance [5]. Children with insulin resistance, in turn, are very likely to develop type 2 diabetes and an adverse lipid profile [6,7]. Moreover, various studies have reported that these individual factors track from childhood into adulthood [8,9]. Hence children with insulin resistance and an adverse lipid profile are at higher risk for CVD in later life.

Although the relation of socioeconomic status (SES) to obesity in childhood is extensively documented, few studies addressed the socioeconomic gradient in lipid and glucose metabolism in childhood. In adolescence, parental education was associated with markers of lipid and glucose metabolism, such as insulin, glucose, high-density lipoproteins (HDL), low-density lipoproteins (LDL), and triglycerides, [6] while other studies found no differences in these markers [7]. In 10 year olds, some studies found no educational disparities in cholesterol, triglycerides and HDL, [10] while others showed greater insulin resistance in children from poorer families and with less educated parents [8]. So far no studies examined the socioeconomic gradient in lipid and glucose metabolism in early childhood.

Socioeconomic inequalities in lipid and glucose metabolism in early childhood can provide useful insights into health inequalities in later life. Therefore, the first objective of this study was to investigate the association of socioeconomic status with the lipid and glucose metabolism in 5–6 year olds. The second objective of this study was to investigate many potential mediating factors in this association. As ethnicity is strongly associated with both SES and the lipid and glucose metabolism, [5] this study was conducted in an ethnic homogeneous sample.

## Methods

This study was part of the Amsterdam Born Children and their Development (ABCD) study, a prospective cohort study from fetal life onwards. Details of this study were described previously [11]. Approval was obtained from the Academic Medical Center Medical Ethical Committee, and the Registration Committee of Amsterdam. All participants gave written informed consent for themselves and their children.

### Study population

**Figure 1:**
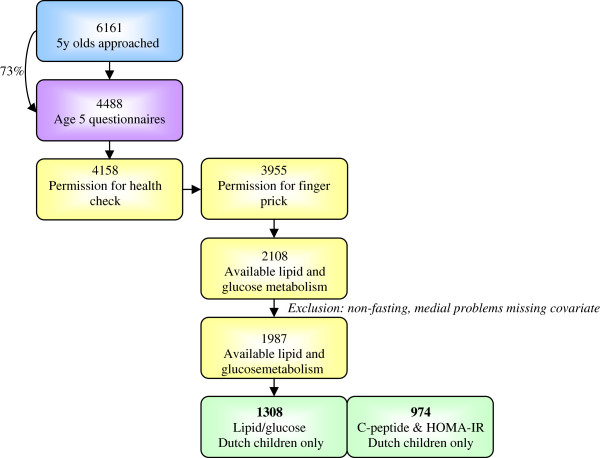
Sampling procedure.

In 2003–2004, 12 373 Amsterdam women who first attended antenatal care were approached to participate. 8266 women returned the pregnancy questionnaire including sociodemographic data, obstetric history, family history, and lifestyle. Of the mothers with a singleton life birth (n = 7863), 6735 gave permission for follow-up (86%). Data on birth outcomes was obtained from Youth Health Care registry. Three months after delivery the mothers received another questionnaire, the infancy questionnaire, concerning i.e. the baby’s feeding (n = 5131). When the children turned five, 6161 mothers received a questionnaire, including an informed consent sheet for a health check of their child (Figure 1). Attrition in follow-up number was largely due to untraceable changes in address or migration. 4488 questionnaires were returned (73%); 4158 gave permission for the age 5 health check and 3955 gave permission for a finger prick. In total, 3321 children were measured during the health check. The finger prick was performed in 2452 children aged five-six.

The present study included only children for which markers of lipid and glucose metabolism were reliably measured (n = 2108) [12]. Additionally, the child should have been fasting since the night before, data on age, sex, height, size at birth, and BMI had to be available. Furthermore, a child with Down syndrome and a child with cystic fibrosis were excluded. Following these criteria, the population was narrowed down to 1987 children. Since there was a small amount of blood obtained and other measures had priority, C-peptide concentrations were available for a smaller number of children. As the lipid and glucose metabolism and educational level differs by ethnicity, [5,13] only children with a Dutch mother and Dutch grandmother were selected for the present study. Thus, 1308 children with complete data were included in the current study’s analyses. A subsample of 974 children had complete data on C-peptide and insulin resistance (HOMA-IR).

### Independent variables

Socioeconomic status (SES) was indexed using maternal education, as education level is the most consistent SES predictor of cardiovascular disease and its risk factors [14]. Maternal education was reported in the childhood questionnaire and was categorized as follows: low (no education or primary school only; lower vocational secondary education or technical secondary education); mid (higher vocational secondary education, intermediate vocational education); high (higher vocational education, university education). In the questionnaire there were 4 categories, but only 5 women reported no education or primary school only, therefore this category was combined with lower vocational secondary education or technical secondary education.

Analyses were repeated with family income adequacy as an indicator of SES to get more insight in the broad construct of SES. Family income adequacy was requested in the childhood questionnaire and was categorized into four categories: (1) inadequate – scored if the mother filled out either “overdraft or in debt” or “using up my savings”; (2) adequate – scored if the mother filled out “can just make ends meet”; (3) bit more than adequate – scored if “can make ends meet and a bit more” was filled out ; and (4) a lot more than adequate – scored if “can make ends meet and a lot more” was filled out.

### Dependent variables

At the age five health check, capillary blood was collected with an ambulatory collection kit (Demecal: Lab Anywhere, Haarlem, The Netherlands) [12]. Fasting glucose (mmol/l), total cholesterol (mmol/l), HDL (mmol/l), LDL (mmol/l), triglycerides (mmol/l), and C-peptide (nmol/l) were determined. 49% of the C-peptide concentrations fell below the detection limit of 0.34 nmol/l. Therefore, associations with C-peptide were explored using survival analysis. Sex, age and BMI of the children were used to predict these concentrations for the missing cases with survival analysis in R (‘survreg’; R2.13.0, R foundation for statistical computing, Vienna, Austria), applying the log-logistic distribution because it was the best fitting, based on log-likelihood. The homeostatic model assessment (HOMA2-IR) was used to quantify insulin resistance, using glucose and C-peptide concentrations [15].

### Covariates

Childhood age (continuous) and sex were included as covariates. Based on the literature, other covariates were considered as potential mediators in the relation between SES and lipid and glucose metabolism. Potential mediators were: size at birth (small-for-gestational age; appropriate-for-gestational age; large-for-gestational age), maternal pre pregnancy body mass index (BMI; kg/m2, continuous), breastfeeding duration (< 1 month, 1–3 months, and > 3 months), height (continuous), childhood BMI (kg/m^2^, continuous), sports club membership (yes/no), walking to school (none, < 30 min a week, 31–60 min a week, > 60 min a week), and cycling to school (none, < 30 min a week, 31–60 min a week, > 60 min a week). Size at birth was defined as small-for-gestational age (SGA) when birth weight was below the 10^th^ percentile of parity and sex specific references of the Perinatal Registration, the Netherlands (PRN), as appropriate-for-gestational age when birth weight was between p10 and p90, and as large-for-gestational age (LGA) when birth weight was greater than the 90^th^ percentile of these references [16]. Birth weight and gestational age were obtained from the Youth Health Care Registration and the PRN. Self-reported pre pregnancy weight and height were used to calculate pre pregnancy maternal BMI. Information on duration of breastfeeding was available from the infancy questionnaire and from the Youth Health Care registration. This prospectively collected information was completed with retrospective information of the 5-year questionnaire to complete the data (19.9% retrospective data). During the health check at age 5, the child’s height was measured to the nearest millimetre using a Leicester height measure (Seca), and weight to the nearest 100 gram using a Marsden weighing scale, model MS-4102. On the basis of these values, BMI was calculated. Dutch sex and age specific references were used in expressing BMI as standard deviation scores [17]. Information on walking and cycling was available from the 5-year questionnaire. Mothers had to fill in how many times their child walked to school and how many times their child walked from school each week. If their child walked to or from school we asked: ‘how many minutes does it take each time?’ The same was done for cycling. The duration of walking and cycling (minutes) per week was calculated by multiplying the frequency per week with the duration each time.

### Statistics

Differences between SES groups were examined using Chi-square tests for categorical variables and ANOVAs for continuous variables (Table 1). Triglycerides and C-peptide were log-transformed as they were positively skewed. In addition, the associations of the lipid and glucose metabolism to all covariates were assessed with linear regression models. First, univariate analyses were performed (Table 2). Second, multivariate analyses were performed including all potential mediators that were associated (p-value < 0.1) with one of the outcome variables in univariate analyses (Table 3). Covariates were considered as potential mediators if they were associated with SES and if they were associated with the lipid and/or glucose metabolism in multivariate analysis [18]. These potential mediators were added in the subsequent models (Table 4) and were considered as mediators if the coefficient of the association between SES and the lipid and/or glucose metabolism decreased > 10% by adjustment for the potential mediator [19]. All statistical analyses were performed using SPSS 15.0 for Windows. A p-value < 0.05 was regarded as significant in all analyses.

**Table 1:** Sample characteristics and metabolic blood profile in means (SD) or percentage (%) by maternal education

	**Total**	**Low education**	**Mid education**	**High education**	**p-value**^**a**^
N	1308	68	216	1024	
Age	5.67 (0.43)	5.66 (0.44)	5.74 (0.44)	5.66 (0.42)	.04
Sex (% boys)	51.1	45.6	48.6	52.1	.42
Height m	1.16 (0.06)	1.17 (0.06)	1.17 (0.06)	1.16 (0.05)	.12
Size at birth					.34
SGA (<p10) (%)	5.4	4.4	8.3	4.9	
AGA	79.9	79.4	78.2	80.3	
LGA (>p90)	14.7	16.2	13.4	14.8	
Pre pregnancy Maternal BMI	22.8 (3.6)	25.1 (4.7)	23.5 (3.8)	22.5 (3.3)	<.001
Breastfeeding duration					<.001
< 1 month (%)	23.1	39.7	31.0	20.3	
1 – 3 months	24.9	30.9	28.7	23.8	
> 3 months	52.0	29.4	40.3	55.9	
Childhood sdsBMI	−0.15 (0.82)	0.14 (1.00)	−0.14 (0.94)	−0.17 (0.77)	.009
Physical activity					
sports club member (% yes)	58.2	52.3	59.0	58.4	.42
Walking to school					.001
No	47.1	47.6	45.1	47.5	
< 30 min a week	23.7	11.1	23.3	24.6	
31 – 60 min a week	19.4	15.9	22.3	19.0	
> 60 min a week	9.7	25.4	9.2	8.9	
Cycling to school					.27
No	62.5	65.6	64.4	61.9	
< 30 min a week	19.5	25.0	16.8	19.7	
31 – 60 min a week	11.4	4.7	13.9	11.3	
> 60 min a week	6.6	4.7	4.8	7.1	
Income adequacy					<.001
Inadequate (%)	8.1	7.5	11.1	7.3	
Adequate	18.9	45.6	27.3	15.4	
Bit more than adequate	41.9	36.8	43.1	42.0	
Lot more than adequate	31.1	5.9	18.5	35.4	
*Outcome*					
Cholesterol (mmol/l)	4.0 (0.7)	4.0 (0.7)	4.1 (0.7)	4.0 (0.7)	.47
HDL-C (mmol/l)	1.3 (0.3)	1.3 (0.3)	1.3 (0.3)	1.3 (0.3)	.99
LDL-C (mmol/l)	2.3 (0.6)	2.3 (0.6)	2.4 (0.7)	2.3 (0.6)	.45
Triglycerides^b^ (mmol/l)	0.6 (0.4 – 0.8)	0.6 (0.5 – 0.8)	0.6 (0.4 – 0.8)	0.6 (0.4 – 0.8)	.66
Glucose (mmol/l)	4.6 (0.5)	4.7 (0.6)	4.6 (0.5)	4.5 (0.5)	.01
n	974	52	154	768	
C-peptide^b^ (nmol/l)	0.32 (0.28 – 0.38)	0.38 (0.32 – 0.44)	0.33 (0.28 – 0.40)	0.32 (0.28 – 0.38)	<.001
HOMA-IR	0.7 (0.3)	0.9 (0.4)	0.8 (0.3)	0.7 (0.2)	<.001

^a^ based on ANOVAs and Chi Square test. P-values for triglycerides and C-peptide were based on log transformed data.

^b^ median (interquartile range).

**Table 2:** Univariate regression analysis (95% CI) of lipid and glucose metabolism with potential mediators

	**Cholesterol**	**HDL-C**	**LDL-C**	**Log(triglycerides)**	**Glucose**	**Log(C-peptide)**	**Insulin resistance**
Age	0.09 (0.00 – 0.18) **	0.10 (0.06 – 0.13) ****	0.14 (0.06 – 0.22)	0.01 (−0.01 – 0.04)	0.14 (0.08 – 0.21) ****	−0.10 (−0.12 - -0.09) ****	−0.15 (−0.19 - - 0.10) ****
Sex (reference: boys)	0.19 (0.12 – 0.26) ****	−0.02 (−0.05 – 0.01)	0.24 (0.17 – 0.30) ****	0.06 (0.04 – 0.08) ****	−0.13 (−0.18 - -0.08) ****	0.02 (0.01 – 0.03) ***	0.01 (−0.02 – 0.05)
Height in meters	−0.33 (−0.99 – 0.33) *	0.15 (−0.15 – 0.45)	−0.03 (−0.66 – 0.60)	−0.01 (−0.19 – 0.18)	1.20 (0.73 – 1.68) ****	−0.13 (−0.25 – -0.00) **	−0.07 (−0.37 – 0.22)
Size at birth (reference: AGA)							
SGA	0.06 (−0.11 – 0.22)	0.08 (0.01 – 0.16) **	−0.04 (−0.19 – 0.12)	0.01 (−0.04 – 0.05)	−0.02 (−0.14 – 0.10)	−0.01 (−0.04 – 0.02)	0.00 (−0.07 – 0.07)
LGA	−0.12 (−0.22 - -0.01) **	−0.04 (−0.09 – 0.01)	−0.10 (−0.20 – 0.00) *	−0.01 (−0.04 – 0.02)	0.01 (−0.06 – 0.09)	0.01 (−0.01 – 0.03)	0.04 (0.00 – 0.09) *
Maternal BMI	0.00 (−0.01 – 0.01)	0.00 (−0.01 – 0.00)	0.01 (−0.00 – 0.02) *	0.00 (−0.00 – 0.00)	0.01 (−0.00 – 0.02)	0.00 (−0.00 – 0.00)	0.00 (−0.01 – 0.00)
Breastfeeding duration (reference: > 3 months)							
< 1 month	−0.02 (−0.11 – 0.07)	−0.04 (−0.08 – 0.00)	0.00 (−0.09 – 0.09)	0.02 (−0.00 – 0.05) *	−0.01 (−0.08 – 0.06)	0.00 (−0.01 – 0.02)	0.00 (−0.04 – 0.04)
1 – 3 months	0.08 (−0.01 – 0.17)	0.01 (−0.03 – 0.05)	0.07 (−0.02 – 0.15)	0.00 (−0.02 – 0.03)	0.00 (−0.06 – 0.07)	0.00 (−0.02 – 0.01)	0.01 (−0.03 – 0.05)
Childhood sdsBMI	0.01 (−0.04 – 0.06)	−0.01 (−0.03 – 0.01)	0.01 (−0.03 – 0.05)	0.01 (−0.01 – 0.02)	0.05 (0.02 – 0.08) ***	0.03 (0.02 – 0.04) ****	0.04 (0.02 – 0.06) ****
Sports club member (reference: yes)	−0.01 (−0.09 – 0.07)	0.01 (−0.03 – 0.04)	−0.02 (−0.09 – 0.05)	0.00 (−0.02 – 0.02)	0.05 (−0.01 – 0.10) *	−0.01 (−0.02 – 0.01)	0.00 (−0.03 – 0.04)
Walking to school (reference: > 60 min a week)							
No	0.09 (−0.02 – 0.21)	−0.02 (−0.07 – 0.03)	0.04 (−0.07 – 0.15)	0.00 (−0.03 – 0.03)	0.01 (−0.07 – 0.09)	0.01 (−0.01 – 0.04)	0.03 (−0.02 – 0.08)
< 30 min a week	0.07 (−0.06 – 0.19)	−0.01 (−0.07 – 0.05)	0.05 (−0.07 – 0.17)	0.00 (−0.04 – 0.03)	0.00 (−0.09 – 0.09)	0.01 (−0.02 – 0.03)	0.01 (−0.05 – 0.06)
31 – 60 min a week	0.05 (−0.08 – 0.18)	−0.04 (−0.10 – 0.02)	0.03 (−0.10 – 0.16)	0.01 (−0.03 – 0.05)	0.06 (−0.03 – 0.16)	0.02 (−0.01 – 0.04)	0.05 (−0.01 – 0.11)
Cycling to school (reference: > 60 min a week)							
No	−0.04 (−0.17 – 0.09)	−0.05 (−0.10 – 0.01)	−0.07 (−0.19 – 0.05)	0.01 (−0.03 – 0.04)	−0.01 (−0.10 – 0.09)	0.00 (−0.02 – 0.03)	−0.02 (−0.07 – 0.04)
< 30 min a week	−0.06 (−0.21 – 0.09)	−0.03 (−0.10 – 0.04)	−0.07 (−0.21 – 0.07)	−0.02 (−0.06 – 0.02)	0.03 (−0.08 – 0.14)	0.00 (−0.02 – 0.03)	−0.02 (−0.09 – 0.05)
31 – 60 min a week	−0.06 (−0.22 – 0.10)	−0.01 (−0.08 – 0.07)	−0.08 (−0.23 – 0.08)	−0.01 (−0.06 – 0.03)	0.02 (−0.10 – 0.14)	0.01 (−0.02 – 0.05)	0.00 (−0.07 – 0.08)

* p < 0.1; ** p <0.05; *** p < 0.01; ****p < 0.001.

**Table 3:** Multivariate linear regression models of glucose, C-peptide, and insulin resistance, including all presented variables

	**Glucose**	**Log(C-peptide)**	**Insuline resistance**
	**β (95% CI)**	**β (95% CI)**	**β (95% CI)**
Maternal education (reference: high)			
Mid	0.07 (0.00 – 0.01)	0.02 (0.00 – 0.04)	0.04 (0.00 – 0.09)
Low	0.14 (0.02 – 0.26)	0.06 (0.03 – 0.08)	0.13 (0.06 – 0.20)
Age	0.10 (0.03 – 0.17)	−0.12 (−0.13 - -0.10)	−0.17 (−0.22 - -0.13)
Sex (reference: boys)			
Girls	−0.12 (−0.18 - -0.07)	0.02 (0.00 – 0.03)	0.01 (−0.02 – 0.64)
Height in meters	0.64 (0.07 – 1.20)	0.16 (0.03 – 0.29)	0.31 (−0.02 – 0.64)
Size at birth (reference: AGA)			
SGA (<p10)	−0.01 (−0.13 – 0.11)	0.02 (−0.01 – 0.04)	0.04 (−0.03 – 0.11)
LGA (>p90)	−0.03 (−0.10 – 0.05)	0.00 (−0.02 – 0.02)	0.02 (−0.02 – 0.07)
Sports club member (reference: yes)	0.03 (−0.02 – 0.09)	0.00 (−0.02 – 0.01)	0.01 (−0.03 – 0.04)
Childhood sdsBMI	0.04 (0.01 – 0.08)	0.03 (0.02 – 0.03)	0.04 (0.02 – 0.06)

**Table 4:** Linear regression models of the association between maternal education and glucose, C-peptide, and insulin resistance

	**Glucose**	**Log(C-peptide)**	**Insulin resistance**
	**β (95% CI)**	**β (95% CI)**	**β (95% CI)**
Model 1^a^			
Maternal education			
Mid	0.07 (−0.002 – 0.14)	**0.02 (0.002 – 0.04)**	**0.05 (0.002 – 0.09)**
Low	**0.16 (0.04 – 0.27)**	**0.07 (0.04 – 0.10)**	**0.15 (0.08 – 0.22)**
Model 2^b^			
Maternal education			
Mid	0.07 (−0.00 - 0.14)	**0.02 (0.002 – 0.04)**	**0.05 (0.002 – 0.09)**
Low	**0.15 (0.03 - 0.27)**	**0.07 (0.04 – 0.09)**	**0.15 (0.08 – 0.22)**
Model 3^c^			
Maternal education			
Mid	0.07 (−0.01 - 0.14)	**0.02 (0.002 - 0.04)**	**0.04 (0.001 – 0.09)**
Low	**0.14 (0.02 - 0.26)**	**0.06 (0.03 – 0.08)**	**0.13 (0.07 – 0.20)**

^a^ Model 1 adjusted for age and sex.

^b^ Model 2 adjusted for age, sex, and height.

^c^ Model 3 adjusted for age, sex, height, and sdsBMI.

In all models high education was considered as reference group.

Bold indicates significance.

## Results

The final sample of 1308 children was comparable to the sample of Dutch children (n = 1598 out of n = 2452) who underwent a finger prick, in terms of maternal educational level (p = 0.13), income adequacy (p = 0.97), and BMI (15.4 vs 15.3 p = 0.35). However these children were slightly younger (5.6 vs 5.7, p = 0.004). Children with available markers of lipid and glucose metabolism tended to have higher educated mothers (9.9 vs 8.2 years of education after primary school), older mothers (32.1 vs 30.2 y), mothers with a lower body mass index (23.0 vs 23.2), and higher birth weight (3503 vs 3360 g) compared to the initial ABCD-cohort.

As presented in Table 1, the children’s mean age in the current sample was 5.67 (SD 0.43). Maternal pre pregnancy BMI, duration of breastfeeding, childhood BMI, and duration of walking to school differed between educational groups.

Concentrations of cholesterol, HDL, LDL, and triglycerides were equal between educational groups. However, there were educational inequalities in fasting glucose, C-peptide, and insulin resistance with higher values in children of less educated mothers (Table 1). In univariate analyses (Table 2), cholesterol, LDL, and triglycerides were associated with sex, with higher levels in girls. In addition, cholesterol and HDL were associated with age, and size at birth. Glucose, C-peptide, and insulin resistance were associated with age and childhood BMI. Moreover, glucose and C-peptide were associated with sex, and height. As sports club membership and size at birth were borderline associated (p < 0.1) with a marker of the glucose metabolism, these variables were included in the multivariate analyses as well (Table 3). In multivariate analyses, the higher the childhood BMI, the higher were glucose, C-peptide, and insulin resistance. Childhood height was associated with glucose and C-peptide as well. Other covariates were not associated with glucose, C-peptide, and insulin resistance in multivariate analyses. Thus, only childhood height and BMI were indicated as a potential mediator and adjusted to the subsequent model.

As can be seen from Table 4, adjustment for childhood height (model 2) to the basic model (model 1) did not decrease the coefficients. Additional adjustment for childhood BMI (model 3) led to a considerable decrease in the coefficients of glucose, C-peptide and insulin resistance among children of low educated mothers. After adjustment for age, height, sex, and BMI, the association between low maternal education and glucose, C-peptide, and insulin resistance remained significant.

### Replicating the analyses using income adequacy as an indicator of SES

The analyses were repeated using income adequacy as an indicator of SES. Income adequacy was not associated with covariates, except maternal BMI and childhood BMI. The lesser the income adequacy, the higher the BMI. Income adequacy was not associated with the lipid and glucose metabolism in analyses adjusted for age, height, and sex (data not shown).

## Discussion

This study sought to investigate the relationship between socioeconomic status and the lipid and glucose metabolism in 5–6 year olds. Interestingly, there were no differences in lipid profile, while there were gradual differences in the glucose profile with higher levels of glucose, C-peptide, and insulin resistance in children with low educated mothers.

So far, the association of SES to lipid and glucose metabolism has not been demonstrated at this young age. In 10 year olds, there were also no SES differences in cholesterol, triglycerides, and HDL [10]. In a slightly older Danish cohort there were no differences in triglycerides and HDL, but there were educational differences in insulin resistance (HOMA-IR) [8]. Among adolescents, Goodman and colleagues showed education related differences in insulin, glucose, insulin resistance (HOMA-IR), and also in HDL and LDL [6]. It appears that there are education related differences in insulin resistance in early childhood and that these differences in dyslipidemia become clear later in life. This concords with the pathogenesis of dyslipidemia of which researchers, as stated by Brunzell and Hokanson, hypothesized that “central obesity causes insulin resistance and elevated free fatty acid levels, with the resultant increase in hepatic apoB secretion and increased hepatic lipase activity leading to hypertriglyceridemia, small dense LDL, and decreased HDL” [19]. Thus, insulin resistance seems to be a cause of dyslipidemia. As children with low educated mothers had higher levels of insulin resistance, they are consequently at risk for dyslipidemia later in life.

### Inequalities explained

Educational inequalities in glucose, C-peptide and insulin resistance were partly explained by standardized child BMI. However, independently of BMI there remained an association between maternal education and the glucose profile. This association can not be explained by maternal pre pregnancy BMI, breastfeeding duration, physical activity, and income adequacy. In addition, the association between maternal education and the glucose metabolism can not be explained by size at birth as size at birth was not associated with markers of the glucose metabolism in multivariate analyses. However, there was a significant association between insulin resistance and LGA in univariate analyses. This finding is comparable to other studies and suggests the overriding importance of current BMI [20,21]. The association of maternal education to glucose, C-peptide, and insulin resistance might be explained by other factors, such as carbohydrate intake, and stress of living. Previous research indicates that insulin resistance is associated with carbohydrate intake [22]. In our sample, there was no association of insulin resistance to carbohydrate intake in a subgroup of 765 children who completed a food frequency questionnaire at age 5–6. In addition, the carbohydrate metabolism and cholesterol metabolism are influenced by cortisol. Low SES children are at risk for dysregulation of cortisol due to stress of living [6,23]. Thus, in low SES children, insulin resistance might be caused by stress of living. For planning preventive initiatives it is important to better understand socioeconomic inequalities in insulin resistance. Therefore, future research should take into account cortisol in particular.

### Strengths and limitations

The results presented here are subject to limitations. First, the current study is conducted in a large prospective cohort study and unfortunately, selective loss to follow-up was present, as in most cohort studies. The current subgroup tends to be a slightly healthier and higher SES reflection of the population. This underrepresentation of low SES children might be the reason that some of the actual associations did not reach significance. The non significant associations were, however, far from statistical significance: even in a population with higher low SES prevalence, the associations are not likely to become significant. Second, because this is a large epidemiological study, insulin resistance was measured with the HOMA model using C-peptide rather than oral glucose tolerance test or euglycemic clamp. Unfortunately, due to the relatively high detection limit, we had many missing on C-peptide and had to impute this variable using survival analysis. We repeated the analyses including only those with original C-peptide data (without imputation). In these analyses the education related differences in C-peptide were slightly larger (0.38 nmol/l vs 0.45 nmol/l for high and low education respectively). We infer that multiple imputation underestimated rather than overestimated our results. Finally, we used two indicators of socioeconomic status, but these indicators cannot capture the whole SES. In addition, income adequacy is a subjective indicator of SES, so an ‘adequate’ income could interpret differential by participants.

## Conclusions

The current study shows relevant educational inequalities in glucose levels and insulin resistance at age 5–6, while there are no educational inequalities in lipid profile. As previous research at adolescence showed educational inequalities in lipid profile, our findings have important implications for understanding risk trajectories among youth and suggest that educational inequalities begin in early childhood and may increase over time as the children become older. The association between maternal education and insulin resistance is partly explained by childhood BMI. However, BMI and various other factors cannot fully explain the educational gradient in insulin resistance. Whilst identification of specific influences underlying the educational gradient in insulin resistance requires further investigation, our findings suggest that reducing BMI decreases the educational gradient in insulin resistance at early childhood and may improve cardiovascular disease risk in later life.

## Abbreviations

95% CI: 95% confidence interval; ABCD: Amsterdam Born Children and their Development; AGA: Appropriate-for-gestational age; ANOVA: Analysis of variance; BMI: Body mass index; HDL: High-density lipoprotein; HOMA: Homeostatic model assessment; LDL: Low-density lipoprotein; LGA: Large-for-gestational age; OR: Odds ratio; PRN: Perinatal Registration: the Netherlands; SES: Socioeconomic status; SGA: Small-for-gestational age.

## Competing interest

The authors declare that they have no competing interests

## Authors’ contributions

GVDB had primary responsibility for data analysis and writing the manuscript. MVE coordinated data collection as project manager of the ABCD study, provided statistical advice and participated in writing the manuscript. TV coordinated data collection as project manager of the ABCD study, provided statistical advice and participated in writing the manuscript. RG, supervised the design of the study, supervised data analysis and writing of the manuscript. All authors have seen and approved this final version.

## Funding

None

## Pre-publication history

The pre-publication history for this paper can be accessed here:

http://www.biomedcentral.com/1471-2458/12/591/prepub
